# Posttraumatic stress disorder among earthquake survivors of the Wenchuan area (Sichuan, China)

**DOI:** 10.3402/ejpt.v5.26531

**Published:** 2014-12-09

**Authors:** Chunlan Hong, Jingming Cao, Thomas Efferth

**Affiliations:** Department of Pharmaceutical Biology, Institute of Pharmacy and Biochemistry, Johannes Gutenberg University, Mainz, Germany

**Keywords:** PTSD, earthquake, biomarker, phytotherapy, intervention

## Abstract

**Background:**

On May 12, 2008, an earthquake with a power of 8.0 M on the Richter scale occurred in the Wenchuan County of Sichuan Province in southwest China, which was unprecedented in magnitude and aftermath. Approximately 70,000 people were killed and nearly 20,000 went missing. The earthquake caused a wide number of mental and physical health outcomes among survivors, and posttraumatic stress disorder (PTSD) was one of the most commonly studied.

**Methods:**

We conducted a systematic overview to assess research achievements about PTSD in the past 6 years after the Wenchuan earthquake, including symptoms and risk factors about PTSD among Wenchuan earthquake survivors, as well as research developments in genetics, molecular biology, and treatment of PTSD.

**Results:**

The large body of research conducted after the Wenchuan earthquake suggests that the burden of PTSD among persons with high exposure was substantial. Adolescents and adults were among the most studied populations with high prevalence rates. Phytotherapy with Chinese herbs as well as acupuncture were rarely studied as of yet, although published data indicated promising therapy effects. Genome-wide microarray technologies are widely used in experimental mice and rat models to study PTSD mechanisms as well as in patients suffering from PTSD and other psychosomatic disorders to search for novel biomarkers and to monitor the effectiveness of treatment interventions.

**Conclusion:**

Using genomic and transcriptomic technologies, our future research will focus on the efficacy and safety of Chinese medicine to find potential interventions and effective treatments of PTSD.

